# Controlled Cohort Study of Serum Gonadal and Adrenocortical Steroid Levels in Males Prior to Onset of Rheumatoid Arthritis (pre-RA): A Comparison to pre-RA Females and Sex Differences among the Study Groups

**DOI:** 10.1155/2013/284145

**Published:** 2013-11-24

**Authors:** Alfonse T. Masi, Azeem A. Rehman, Robert T. Chatterton, Huaping Wang, Ned J. Goertzen, Kevin B. Elmore, Jean C. Aldag

**Affiliations:** ^1^Medicine and Epidemiology, College of Medicine (UICOMP), University of Illinois, Peoria, IL 61656, USA; ^2^Department of Medicine, UICOMP, One Illini Drive, Peoria, IL 61656, USA; ^3^University of Illinois College of Medicine at Peoria (UICOMP), Peoria, IL 61656, USA; ^4^Northwestern University, Chicago, IL 60611, USA

## Abstract

Serum testosterone levels are generally reported to be lower in male rheumatoid arthritis (RA) patients, but it is not determined if a deficiency may occur before clinical onset of disease (pre-RA). Lower testosterone levels were recently reported in males many years before RA onset but were predictive only of rheumatoid factor (RF)—negative disease. A preceding prospective study did not reveal androgenic-anabolic hormone association with risk of RA in men or women. This cohort study of males analyzed baseline serum levels of gonadal and adrenocortical steroids, luteinizing hormone, and prolactin in 18 pre-RA versus 72 matched non-RA control (CN) subjects. Findings in males were compared to those in female pre-RA and CN subjects in the same cohort, and sex differences were analyzed. Steroidal and hormonal levels, including total testosterone, were similar between male study groups. In females, mean (±SE) serum androstenedione (nmol/L) was slightly (*P* = 0.048) lower in 36 pre-RA (6.7 ± 0.36) than 144 CN (7.6 ± 0.22). With the exception of 3 partial correlations of hormonal variables observed to differ between pre-RA versus CN subjects, the patterns were similar overall. However, partial correlations of hormonal variables differed frequently by sex, both within and between study groups.

## 1. Introduction

 The onset of rheumatoid arthritis (RA) occurs about 5-fold more frequently in women of child-bearing ages than among male counterparts [1]. Early age at menopause (≤45 yrs) was recently found to be associated with the subsequent risk of developing RA [2]. Such findings suggest that sex hormones may influence predisposition to this disease in women. In male RA patients with active disease, testosterone levels are reported to be lower than those in healthy control (CN) subjects [3, 4]. However, it is not known if such hormonal alteration results from inflammatory manifestations of active clinical disease or if it may be a preexisting risk factor before clinical onset (pre-RA).

Recently, testosterone levels were reported to be lower in males many years before RA onset, as identified in a large Swedish cohort [5]. However, a significant association of lower testosterone levels was predictive only for the minority subset of patients having negative rheumatoid factor (RF-negative) disease [5]. A preceding retrospective case-control nested study within a large Finnish cohort did not find baseline serum total testosterone or dehydroepiandrosterone sulfate (DHEAS) levels to be predictive of the subsequent onset of RA, either in 32-male or in 84-female cases [6].

Our prospective cohort of male subjects previously revealed a cluster excess (*P* = 0.044) of combined baseline relatively lower serum total testosterone (<15 nmol/L) and lower cortisol (<140 nmol/L) levels in 2 (11.1%) of 18 pre-RA cases, but only in 1 (1.4%) of 72 matched CN subjects [7]. Serum levels of either testosterone or cortisol alone did not differ between the male pre-RA versus CN subjects in the prospective study [7].

 In women, relative insufficiency of adrenocortical and gonadal hormones is suspected to predispose to risk of developing RA as a component of neuroendocrine immune (NEI) mechanisms [7–9], in addition to other multifactorial genetic and environmental pathogenesis [10, 11]. Lower androgenic-anabolic (AA) steroid levels were reported to occur before the onset of RA in a minority subset of women, particularly when the disease manifested in premenopausal ages [7, 8]. However, such AA steroid relations, like lower dehydroepiandrosterone sulfate (DHEAS) levels, were not observed in the two other cohort studies [6, 12]. No prospective study report had previously analyzed sex differences in hormonal profiles of pre-RA versus CN subjects, which are also analyzed.

This cohort study investigated a broad panel of baseline serum gonadal and adrenocortical steroids, luteinizing hormone (LH), and prolactin (PRL) in male pre-RA versus matched non-RA cohort control (CN) subjects and compared the findings in males to those in female pre-RA and CN subjects. Sex differences in partial correlations of steroidal and hormonal variables were analyzed within and between the study groups.

## 2. Materials and Methods

### 2.1. The RA Precursors Study (RAPS) Database at This Institution

The RA Precursors Study (RAPS) was initiated at this institution in 1991 [9]. Baseline personal data and serum samples from the pre-RA cases and matched CN cohort subjects were donated by “Operation CLUE I,” a community-wide prospective study [14–16]. The CLUE I 1974 entry cohort had enrolled 8,680 males and 12,381 females of Washington County, Maryland, USA. The RAPS database currently includes 90 males (18 pre-RA and 72 CN) and 180 females (36 pre-RA and 144 CN) study subjects, in a ratio of 1 pre-RA: 4 CN. The UICOMP Institutional Review Board has approved this research for assurance of confidentiality. 

All pre-RA cases in this study conform to The European League Against Rheumatism (EULAR) recommendations [17]. The sole rheumatologist in the cohort community diagnosed and confirmed the RA cases according to the American College of Rheumatology (ACR) 1987 revised classification criteria [18]. Clinical onsets of RA in our male and female cases occurred 3 to 20 years following the 1974 entry cohort (1977 to 1994), after a median of 12 years [9]. No matched comparison subject had a diagnosis of RA in the community rheumatologist's practice. The non-RA cohort CN subjects were matched to pre-RA cases on sex, race (all Caucasians), and usually within one year of age at entry. The selected CN were the closest in chronological sequence of enrollment in the cohort to the pre-RA, analogous to another case-control study [19].

In 1992, funding had become available to begin the hormonal assays. The first set of baseline pre-RA females (*n* = 14) and their 4 matched cohort CN (*n* = 56) subjects were identified in the cohort. Their frozen sera were sent from CLUE I to the Northwestern University (NWU) reference laboratory [9, 13]. As additional funding became available in 1994, the second set of female pre-RA (*n* = 22) and CN (*n* = 88) subjects were identified and their frozen sera were sent from CLUE I to the NWU laboratory [9, 13]. With additional funding in 1996, the male pre-RA (*n* = 18) and CN (*n* = 72) subjects were identified and their frozen sera were sent from CLUE I to the NWU laboratory [9]. All baseline stored (−70°C) cohort sera were analyzed in matched sets of 1 pre-RA and 4 CN, without knowledge of subject status.

### 2.2. Assay Methods Were Developed for a Comprehensive Panel of Serum Steroids (Figure 1)

**Figure 1:**
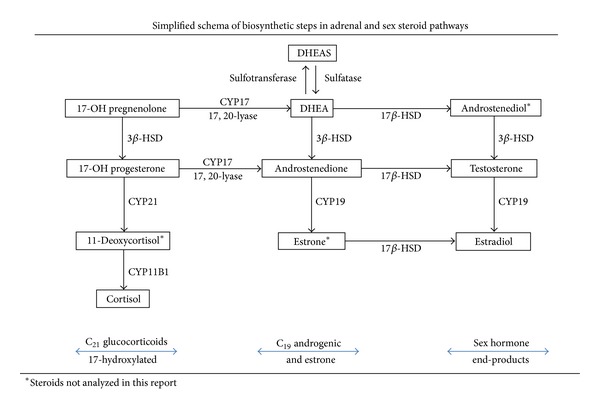


A comprehensive panel of adrenal and sex steroids was assayed in males and females (Figure 1), using the previously developed and described methodology [13]. The pituitary hormones (PRL, LH) were assayed by ELISA techniques [20, 21]. Intra-assay percentile coefficients of variation (% CVs) were all less than 12%, as the measurement criterion for acceptability. Too few batches of assays were performed in the 1992 or 1994 sets to analyze inter-assay variability [13].

Since the female sera were assayed in separate 1992 and 1994 batches, the steroid and hormonal results of the smaller number of first set samples were normalized by their mean values to the means of the larger second set samples [13]. The male sera were assayed completely in 1996 [9], and those values were analyzed as reported without normalization. The assayed steroidal profile in women was larger than that in males, including the majority of the non-17-hydroxylated steroids (mineralocorticoid pathway), as previously reported [13]. Accordingly, a minority of female subjects had insufficient sera to perform the full panel of the other hormonal assays [13] performed in the males (Figure 1). Hence, the minority of missing steroid values in females were multiply imputed, as described below. In both sexes, assay priority was given to cortisol, DHEAS, luteinizing hormone (LH), and prolactin (PRL), which were completely assayed in male and female subjects.

### 2.3. Statistical Methods

In males, sera were sufficient for total assays of the sex hormones, total testosterone (T) and estradiol (E2), their C19 androgenic steroid precursors (DHEA and androstenedione), cortisol, their C21 17-hydroxylated glucocorticoid (GC) precursors (17-OH pregnenolone and 17-OH progesterone), and the pituitary hormones (LH and PRL). In females, sera were sufficient for total assays of cortisol, DHEAS, and the pituitary hormones, but not for the other steroids. The multiple imputation (MI) technique incorporates acceptable values into the data set for those that are assumed to be missing at random [22, 23]. The MI technique was performed using the SAS 9.2 Software (SAS Institute Inc., Cary, NC) [24]. A Markov chain Monte Carlo (MCMC) method was selected when conducting MI with SAS [24], since the data are assumed to have an arbitrary missing data pattern. Then, 10 imputed data sets for each variable were systematically analyzed to derive a single mean value for each of the variables with missing entries, using the IBM SPSS 21.0.0.0 (IBM SPSS, 2012) program AGGREGATE [25] for subsequent analyses. Frequency distributions of the imputed versus the originally reported values were always closely similar for each variable. 

A ratio of the sex steroids, E2 (pmol/L) : T (nmol/L) × (10^3^), was created as an indicator of the physiological balance of these hormones. The ratio is increased with body mass in men [26] but may be decreased among women with polycystic ovary syndrome [27]. A family history of RA in a first-degree relative (FDR) was reported in 6 (33.3%) of the 18-male and 5 (13.9%) of the 36-female cases and was used as a stratification variable [28]. That designation was randomly assigned to the CN subjects in proportion to the population age and sex distribution [28]. A baseline rheumatoid factor (RF) stratification variable was also created. The total cohort was divided into quadrants of baseline (1974) lower versus higher median values of both IgA and IgM isotype RF levels [29]. In a logistic regression (LR) model (data not provided), the higher than median combined IgA/IgM RF results (+IgM and +IgA = yes, in quadrant 4) versus other values significantly (*P* = 0.006) predicted the dependent RA (versus CN) outcome, OR 3.16 (1.381–7.225). The variable of the combined higher quadrant 4 (yes) versus the other quadrants 1–3 (no) was employed to stratify the subjects into assigned baseline RF-positive versus RF-negative subgroups, respectively.

Natural log conversion was performed on all steroidal and hormonal values to improve their distributions for statistical analysis. The analyzed variables were acceptable in unimodality and symmetry, after elimination of extreme outliers. Such outliers were observed in several hormones, as expected in physiological peaks of E2 during ovulatory surges, whereas lowest values may be observed during the luteal phase and in postmenopausal women. The extreme outliers were assigned (Winsorized) to the upper ranges observed in the population frequency distributions [30].

Partial correlations of the full panel of individual steroids, E2/T ratio, LH, and PRL were performed on the natural log-converted values, which were age- and sex-adjusted, as appropriate. The correlational analyses were performed in order to search for possible differences in hormonal interactions between study groups. The significance of differences in partial correlations of hormonal levels between the pre-RA versus CN groups was estimated by the Fisher *r*-to-*z* transformation [31, 32]. When a significant difference was found in correlations between study groups by the *r*-to-*z* method, multivariate regression analysis (MRA) models were employed to validate the individual partial correlation (*r*_*p*_) results, within each study group or between sexes. The MRA model allows the investigator to control a number of additional variables, like potential confounders, to more accurately determine the relationship between the two primary variables of interest. The standardized *β* correlations derived from the MRA were again used in the Fisher *r*-to-*z* transformation [31, 32]. In the MRA models, one member of the correlational pair was selected as the independent predictor, usually the physiological precursor. The counterpart partial correlational member was then assigned as the dependent (outcome) variable in the models. The following additional independent variables were entered in the models: (1) CLUE cohort entry age of subjects; (2) sex, as appropriate; (3) a 7-point gradient score in history of cigarette smoking (cig7); (4) interval in hours (HR) from the last meal until blood sample donation; and (5) interval in years from cohort entry (1974) until the pre-RA case had clinical onset of disease (CLUE to RA) (similarly assigned to the matched CN subjects within sets). In this exploratory study, a significance level of *P* ≤ 0.050 was accepted without adjustment for multiple comparisons [33].

## 3. Results and Discussion

### 3.1. Comparison of Steroidal and Hormonal Values of Study Groups by Sex (Table 1)

 In males, the 18 pre-RA and 72 matched CN subjects had similar mean levels of their serum gonadal and adrenocortical steroids, the estradiol (E2)-to-testosterone (T) ratio, luteinizing hormone, and prolactin, which did not differ significantly by *t*-test analysis (Table 1). Serum total testosterone levels were closely similar between the study groups in both males and females. The mean testosterone values were also closely similar between male and female study groups, when stratified by either a history of RA in a first degree relative (FDR) or by baseline categories of upper-versus-lower median levels of rheumatoid factor isotypes (IgA and IgM).

In females, the steroid and hormonal values were also similar in the study groups. However, the mean (±SE) androstenedione level was slightly (*P* = 0.048) lower in the 36 pre-RA (6.7 ± 0.36) than in the 144 CN (7.6 ± 0.22), as was recently reported using our data set of originally reported assays [13], before imputation. 

### 3.2. Partial Correlations of Assayed Values by pre-RA versus CN Study Groups in Males (Table 2)

 The panel of steroids and pituitary hormone levels were correlated separately in the male pre-RA (top) versus CN (bottom) subjects, using the age-adjusted partial correlation method (Table 2). The matrix patterns were overall similar. A single difference (Δ*P* = 0.034) was observed between stronger positive correlation of 17-hydroxypregnenolone (OH-Preg) and cortisol in the 18 pre-RA (*r*_*p*_ = 0.843, *P* < 0.001) than in the 72 CN (*r*_*p*_ = 0.557, *P* < 0.001). The difference was validated in a multivariate regression analysis (MRA) model (Δ*P* = 0.002), including relevant independent covariates, as specified in Statistical Methods.

### 3.3. Partial Correlations of Values by pre-RA versus CN Study Groups in Females (Table 3)

 Among females, the correlational patterns were also similar overall between the 36 pre-RA and 144 CN subjects (Table 3). However, two low-level correlational differences (Δ*P* = 0.022 and Δ*P* = 0.033) were observed between study groups. In both, the pre-RA correlations were positive, whereas they were, respectively, negative in the CN, as follows: androstenedione with the E2/T ratio (*r*_*p*_ = 0.323, *P* = 0.063 versus *r*_*p*_ = −0.113, *P* = 0.181) and T with prolactin (*r*_*p*_ = 0.207, *P* = 0.232 versus *r*_*p*_ = −0.199, *P* = 0.017). The correlational differences (r-to-z) were validated in MRA models, *P* = 0.032 and *P* = 0.029, respectively (Table 3), as specified in Statistical Methods.

### 3.4. Partial Correlations of Values by pre-RA versus CN Study Groups in Total Subjects (Table 4)

Among the total 54 pre-RA versus 216 CN subjects, the sex- and age-adjusted partial correlational patterns were again similar overall (Table 4). Only one of the two low-level differences observed in females persisted in the total subjects. Again, the correlation of androstenedione with the E2/T ratio was weakly positive in pre-RA (*r*_*p*_ = 0.242, *P* = 0.087), but negative in CN (*r*_*p*_ = −0.090, *P* = 0.191). This difference (Δ*P* = 0.034) was validated in the MRA model (*P* = 0.015), as specified in Statistical Methods.

### 3.5. Correlations of pre-RA versus CN by Sex, and of Males versus Females by Study Groups (Table 5)

The top three entries in Table 5 summarize the above-described differences (*Z*s) between pre-RA versus CN subjects in their respective partial correlations observed in males (Table 2) and in females (Table 3). The respective correlational differences (*Z*s) between study groups (for CN-preRA) and their *P* values are bolded. The 3 correlational differences (*Z*s) observed between pre-RA versus CN, in either males or females (Table 5, top), also differed significantly (*P* ≤ 0.010) between the sexes (Δ*Z*s, for sex), as shown in the last two columns. Their sex differences in *Z* values were evaluated on a probability distribution, and the significance levels are indicated by superscript symbols for 3 levels as well as exact values (Table 5).

In the correlation of adione with the E2/T ratio, the study group difference (*Z* for CN-pre-RA) was significantly negative in females (*Z* = −2.287, *P* = 0.022) but was slightly positive in males (*Z* = 1.571, *P* = 0.116). The preceding sex difference in *Z* values of those CN-pre-RA partial correlations between males and females (Δ*Z* of −3.858) was highly (*P* < 0.001) significant (Table 5, last column symbol). The basis of that sex difference (Δ*Z*) was derived essentially from the opposite correlations in the pre-RA males (*r*_*p*_ = −0.377, *P* = 0.136) versus females (*r*_*p*_ = 0.323, *P* = 0.063), yielding that respective r-to-z probability difference (*P* = 0.019) for the pre-RA group.

This difference in correlations of the 18 male minus the 36 female (ΔM-F) pre-RA cases is also entered in the 1st row of the left lower section of Table 5. That complete row also indicates a stronger (*P* = 0.020) correlation of adione with the E2/T ratio in female pre-RA (*r*_*p*_ = 0.323) versus CN (*r*_*p*_ = −0.113). The analogous correlation of adione with E2 in the female pre-RA (*r*_*p*_ = 0.502) versus CN (*r*_*p*_ = 0.173) was not quite (*P* = 0.051) significantly different (Table 5).

Correlations of steroids and hormones are expected to differ physiologically between the sexes, even when adjusted for age. Accordingly, numerous sex differences were observed in the panel of correlations within the 54 pre-RA and within the 216 CN subjects, as indicated in the lower section of Table 5, stratified by the pre-RA versus CN groups. Significant correlational differences between sexes were bolded when exclusively observed either in pre-RA or in CN subjects, but not in both groups. 

The first 3 entries in the lower panel (Table 5) indicate those correlations that differed by sex only in the total 54 pre-RA; and their *Z* and *P* values are bolded. The subsequent 15 entries are the total correlations which differed by sex in the 216 CN subjects, 9 of which occurred exclusively in that larger sample and those values are bolded accordingly. Six partial correlations in the listings were significantly different by sex in both pre-RA and CN study groups and are not bolded. Thus, 3 of the 9 sex differences observed in the pre-RA were significant only in that group, and 9 of the 15 sex differences in the CN were significant only in those subjects. In addition, two significant sex differences were observed only in the total 270 subjects and are not included in Table 5. Those low level sex differences in partial correlations were LH with E2 and PRL with 17-OH progesterone. The remainder of the sex differences observed in total subjects almost entirely reproduced results of the 216 CN group (Table 5).

The sex differences in correlations (*Z*s for ΔM-F) observed within the 54 pre-RA and within 216 CN subjects (Table 5, bottom) were tested for additional significance of their differences (Δ*Z*s for sex). The sex differences between the pre-RA versus CN study groups (Δ*Z*s for M-F) were particularly strong (*P* < 0.001) for 4 correlations (Table 5). Three of the correlations involved androstenedione (adione). The main contributor to the sex difference of the adione correlation with the E2/T ratio was derived from pre-RA subjects; they had a negative correlation in males and a positive correlation in females (*Z* = −2.338, *P* = 0.019). The other marked (*P* < 0.001) sex differences was derived mainly from the CN group.

In the adione correlation with E2, the association was stronger in male CN versus pre-RA, but the reverse occurred in females. An analogous differential was observed for the adione correlation with coritsol, which also was derived mainly from the CN subjects. Both androstenedione and cortisol levels were recently found to be relatively deficient in a minority subgroup of pre-RA women, as recently reported [13, 34]. The fourth marked sex difference in correlations between pre-RA versus CN occurred in PRL versus DHEAS (Table 5). It also was derived mainly from the CN group, which had a stronger male than female association, not observed in the pre-RA subjects. In each of the preceding marked sex differences, the male pre-RA had a less positive or negative correlation with the counter-part hormone than was observed in the CN subjects. In females, stronger correlations of adione with the paired hormone were observed in the CN versus pre-RA, being significant with the E2/T ratio (*P* = 0.020) and nearly so (*P* = 0.0512) with E2.

An overall interpretation of sex differences (ΔM-F) between the total pre-RA versus CN study subjects is challenging. Those findings are indicated by the *Z* score differences (delta *Z*s), shown in the next to last column of the lower section of Table 5. All 8 significant delta *Z* values have a negative sign (*P* = 0.036), derived from subtracting the *Z* scores of ΔM-F of the 216 CN from the respective *Z* scores of the 54 pre-RA. Two of the 8 significantly negative delta *Z* values were derived mainly from the stronger correlation coefficients of the 36 female than the 18 male pre-RA cases (left panel of entries). In contrast, the remaining 6 significantly negative delta *Z* scores derived mainly from the stronger correlation coefficients of the 72 male versus 144 female CN subjects (right panel of entries), than the corresponding *Z* scores of the ΔM-F derived from the total 54 pre-RA cases. The data may suggest that selected hormonal correlations could be stronger in female than male pre-RA, whereas the reverse may occur in control subjects.

## 4. Conclusions

A large panel of serum gonadal and adrenocortical steroids, LH, and PRL was analyzed in males prior to onset of rheumatoid arthritis (pre-RA) in comparison to matched cohort control (CN) subjects. Results in males were compared to findings in pre-RA and CN females, derived from the same cohort. Sex differences in the steroidal and hormonal correlational patterns were also analyzed within and between each study group. 

The only absolute difference observed between pre-RA versus CN subjects was a slightly (*P* = 0.048) lower mean androstenedione level in the female cases. Serum testosterone levels were similar between study groups, in both males and females. When stratified by sex, limited low-level hormonal correlational differences were observed between pre-RA versus CN groups. In the males and females, they included 17-hydroxypregnenolone with cortisol in males, and androstenedione with the E2/T ratio as well as prolactin with testosterone in females. Numerous sex differences in hormonal correlational patterns were observed exclusively in either the pre-RA (*n* = 3) or the CN (*n* = 9) groups or were found in both (*n* = 6) study groups. 

The only study group correlational difference (*P* = 0.033) involving testosterone was observed in females, namely, a positive association with prolactin in pre-RA (*r*_*p*_ = 0.207, *P* = 0.232) and a negative association in CN (*r*_*p*_ = −0.199, *P* = 0.017). The latter correlations were equivalently (*P* = 0.660) negative in the male study groups (Table 5). In this cohort study of modest sample sizes of male and female pre-RA cases, a limited number of hormonal differences were observed between pre-RA versus CN subjects (Table 5, top). Further prospective study of gonadal and adrenocortical hormones is needed in both males and females to determine if absolute differences in levels or correlational patterns may be identified as being confidently associated with the subsequent risk of clinical disease onset.

## Figures and Tables

**Table 1:** Hormonal values in males of reported assays and normalized, imputed values in females to full sample sizes.

Hormones assayed and statistical values	Reported assay values in males			Normalized and imputed values in females		
	pre-RA (*n* = 18)	Control (*n* = 72)	Total (*n* = 90)	pre-RA (*n* = 36)	Control (*n* = 144)	Total (*n* = 180)
17-OH Pregnenolone:						
Mean (nmol/L) ± SE	11.8 ± 1.1	11.9 ± 0.8	11.9 ± 0.7	6.8 ± 0.6	6.8 ± 0.3	6.8 ± 0.3
Median; IQR	11.0; 8.7–14.6	10.2; 7.3–15.2	10.2; 7.7–15.0	5.7; 4.2–8.8	5.7; 4.4–7.9	5.7; 4.4–8.2
17-OH Progesterone:						
Mean (nmol/L) ± SE	5.0 ± 0.5	5.0 ± 0.3	5.0 ± 0.3	4.3 ± 0.5	4.3 ± 0.3	4.3 ± 0.3
Median; IQR	4.5; 3.5–6.0	4.7; 3.1–6.3	4.7; 3.2–6.2	3.6; 1.7–6.1	3.0; 1.9–5.7	3.1; 1.9–5.9
Dehydroepiandrosterone:						
Mean (nmol/L) ± SE	7.6 ± 1.0	8.5 ± 0.6	8.4 ± 0.5	17.9 ± 1.9	18.6 ± 1.0	18.5 ± 0.9
Median; IQR	7.7; 3.9–9.7	7.2; 4.8–10.3	7.2; 4.7–10.1	14.8; 11.5–19.2	15.9; 12.5–20.8	15.6; 12.4–19.7
Androstenedione:						
Mean (nmol/L) ± SE	2.2 ± 0.2	2.2 ± 0.1	2.2 ± 0.1	6.7 ± 0.4	7.6 ± 0.2*	7.5 ± 0.2
Median; IQR	2.2; 1.4–3.0	2.2; 1.5–2.9	2.2; 1.5–2.9	6.6; 5.3–8.3	7.2; 6.2–8.8	7.1; 6.0–8.6
Testosterone (T):						
Mean (nmol/L) ± SE	19.2 ± 1.8	18.3 ± 0.8	18.5 ± 0.8	2.4 ± 0.18	2.5 ± 0.1	2.5 ± 0.1
Median; IQR	17.8; 13.3–24.3	17.4; 13.7–23.3	17.8; 13.6–23.5	2.3; 1.8–2.9	2.3; 1.8–2.9	2.3; 1.8–2.9
Estradiol (E2):						
Mean (pmol/L) ± SE	69.7 ± 6.0	65.4 ± 2.9	66.2 ± 2.6	251.0 ± 60.7	229.5 ± 18.2	233.0 ± 19.7
Median; IQR	68; 49.0–84.3	66.0; 48.0–80.3	66.0; 48.0–81.0	168; 83–276	176; 80–278	173; 81–278
E2/T ratio (×10^3^)						
Mean ± SE	4.1 ± 0.5	4.1 ± 0.3	4.6 ± 0.2	134.0 ± 36.1	123.0 ± 16.5	125.2 ± 15.0
Median; IQR	4.0; 2.7–4.9	3.4; 2.5–5.0	3.5; 2.6–5.0	76.5; 27.7–151	80.2; 37.6–138	78.8; 35.4–138
Cortisol:						
Mean (nmol/L) ± SE	272.8 ± 35.4	291.1 ± 18.7	285.5 ± 16.7	245.2 ± 24.6	233.4 ± 11.8	236.8 ± 10.8
Median; IQR	257; 161–381	255; 186–382	255; 184–369	241; 143–329	205; 155–285	216; 155–286
DHEA sulfate (DHEAS):						
Mean (*μ*mol/L) ± SE	7.5 ± 1.1	7.3 ± 0.4	7.3 ± 0.4	2.5 ± 0.3	3.0 ± 0.2	2.9 ± 0.1
Median; IQR	6.2; 3.8–11.0	6.8; 4.8–8.8	6.8; 4.6–8.9	2.3; 1.5–3.5	2.6; 1.6–3.9	2.5; 1.6–3.8
Luteinizing Hormone (LH):						
Mean (IU/L) ± SE	5.8 ± 0.7	5.9 ± 0.3	5.9 ± 0.3	23.5 ± 3.4	23.4 ± 1.8	23.4 ± 1.6
Median; IQR	5.5; 4.0–7.2	5.1; 4.0–6.9	5.1; 4.0–7.0	19.6; 4.3–36.2	15.7; 5.4–39.5	17.1; 5.3–37.9
Prolactin (PRL):						
Mean (*μ*g/L) ± SE	8.0 ± 1.0	7.0 ± 0.4	7.2 ± 0.4	11.0 ± 0.8	11.9 ± 0.5	11.7 ± 0.4
Median; IQR	7.6; 4.0–11.4	6.1; 4.6–9.0	6.3; 4.6–9.7	9.5; 8.1–13.3	10.4; 8.0–13.4	10.1; 8.1–13.4
Mean ages ± SEs	41.6 ± 2.1	41.8 ± 1.1	41.8 ± 0.9	43.8 ± 2.0	43.9 ± 1.0	43.9 ± 0.9

SE: standard error of mean, IQR: interquartile range of median.

*P = 0.048, as previously reported [13].

**Table 2:** Age-adjusted partial correlations of log-transformed hormones in male pre-RA (top) and CN (bottom)*.

	E2/TEST	OH-PREG	OH-P4	DHEA	ADIONE	TEST	E2	Cortisol	DHEAS	LH	PRL
E2/TEST											
*r*_*p*_		−0.143	−0.355	−0.387	−0.377	−0.813	0.339	−0.234	0.127	0.163	0.404
*P*		0.583	0.162	0.125	0.136	0.000	0.183	0.366	0.627	0.532	0.108
*n*		18	18	18	18	18	18	18	18	18	18
OH-PREG											
*r*_*p*_	−0.211		0.644	0.598	0.355	0.336	0.316	0.843*	0.076	0.056	0.120
*P*	0.077		0.005	0.011	0.162	0.188	0.216	**0.000**	0.773	0.830	0.647
*n*	72		18	18	18	18	18	18	18	18	18
OH-P4											
*r*_*p*_	−0.281	0.724		0.440	0.559	0.539	0.267	0.496	−0.227	0.063	−0.123
*P*	0.018	0.000		0.077	0.020	0.026	0.299	0.043	0.380	0.811	0.640
*n*	72	72		18	18	18	18	18	18	18	18
DHEA											
*r*_*p*_	−0.085	0.605	0.448		0.641	0.543	0.312	0.737	0.344	−0.211	−0.187
*P*	0.482	0.000	0.000		0.006	0.024	0.223	0.001	0.177	0.417	0.472
*n*	72	72	72		18	18	18	18	18	18	18
ADIONE											
*r*_*p*_	0.051	0.507	0.611	0.651		0.467	0.129	0.471	−0.207	0.263	−0.202
*P*	0.670	0.000	0.000	0.000		0.059	0.621	0.057	0.426	0.307	0.437
*n*	72	72	72	72		18	18	18	18	18	18
TEST											
*r*_*p*_	−0.718	0.420	0.508	0.278	0.293		0.228	0.454	−0.242	−0.073	−0.327
*P*	0.000	0.000	0.000	0.019	0.013		0.379	0.067	0.349	0.781	0.200
*n*	72	72	72	72	72		18	18	18	18	18
E2											
*r*_*p*_	0.427	0.283	0.276	0.252	0.443	0.297		0.404	−0.052	0.152	0.141
*P*	0.000	0.017	0.020	0.034	0.000	0.012		0.108	0.842	0.561	0.588
*n*	72	72	72	72	72	72		18	18	18	18
Cortisol											
*r*_*p*_	0.028	**0.557**	0.433	0.538	0.638	0.161	0.256		0.308	0.060	0.086
*P*	0.817	**0.000**	0.000	0.000	0.000	0.179	0.031		0.229	0.818	0.741
*n*	72	72	72	72	72	72	72		18	18	18
DHEAS											
*r*_*p*_	0.220	−0.090	−0.151	0.443	0.132	−0.206	0.049	−0.005		−0.424	−0.040
*P*	0.065	0.456	0.209	0.000	0.274	0.085	0.682	0.967		0.090	0.879
*n*	72	72	72	72	72	72	72	72		18	18
LH											
*r*_*p*_	−0.040	0.213	0.261	0.133	0.103	0.148	0.196	0.217	0.008		−0.024
*P*	0.740	0.075	0.028	0.268	0.394	0.219	0.102	0.069	0.947		0.926
*n*	72	72	72	72	72	72	72	72	72		18
PRL											
*r*_*p*_	0.172	−0.120	−0.181	0.070	−0.070	−0.211	−0.022	0.020	0.416	−0.028	
*P*	0.152	0.320	0.130	0.563	0.563	0.078	0.856	0.868	0.000	0.819	
*n*	72	72	72	72	72	72	72	72	72	72	

*P = 0.034 (*r*-to-*z* method, validated as *P* = 0.002, in the MRA model, as specified in Section 2.3).

**Table 3:** Age-adjusted partial correlations of normalized imputed hormones (logs) in female pre-RA (top) and CN (bottom)*.

	E2/test	OH-PREG	OH-P4	DHEA	ADIONE	Test	E2	Cortisol	DHEAS	LH	PRL
E2/test											
*r*_*p*_		0.213	0.473	0.11	0.323*	−0.718	0.777	0.219	0.124	−0.332	−0.241
*P*		0.227	0.005	0.535	**0.063**	0.000	0.000	0.214	0.486	0.055	0.170
*n*		36	36	36	36	36	36	36	36	36	36
OH-PREG											
*r*_*p*_	−0.061		0.619	0.787	0.624	0.034	0.224	0.075	0.395	−0.071	0.012
*P*	0.473		0.000	0.000	0.000	0.846	0.196	0.667	0.019	0.686	0.943
*n*	144		36	36	36	36	36	36	36	36	36
OH-P4											
*r*_*p*_	0.229	0.704		0.342	0.439	−0.397	0.354	−0.239	0.220	−0.326	0.207
*P*	0.006	0.000		0.044	0.008	0.018	0.037	0.167	0.204	0.056	0.234
*n*	144	144		36	36	36	36	36	36	36	36
DHEA											
*r*_*p*_	−0.231	0.620	0.377		0.685	0.187	0.151	0.224	0.563	0.001	−0.151
*P*	0.006	0.000	0.000		0.000	0.281	0.385	0.195	0.000	0.994	0.388
*n*	144	144	144		36	36	36	36	36	36	36
ADIONE											
*r*_*p*_	**−0.113**	0.433	0.329	0.770		0.070	0.502	0.199	0.632	0.020	−0.069
*P*	**0.181**	0.000	0.000	0.000		0.689	0.002	0.251	0.000	0.908	0.693
*n*	144	144	144	144		36	36	36	36	36	36
Test											
*r*_*p*_	−0.778	0.231	−0.074	0.377	0.296		−0.191	0.057	−0.001	0.302	0.207^†^
*P*	0.000	0.006	0.378	0.000	0.000		0.272	0.744	0.996	0.078	**0.232**
*n*	144	144	144	144	144		36	36	36	36	36
E2											
*r*_*p*_	0.548	0.169	0.350	0.088	0.173	0.017		0.115	0.094	−0.364	−0.105
*P*	0.000	0.044	0.000	0.296	0.039	0.839		0.518	0.591	0.032	0.547
*n*	144	144	144	144	144	144		35	36	36	36
Cortisol											
*r*_*p*_	−0.118	0.163	−0.103	0.173	0.040	−0.001	−0.196		−0.080	−0.092	−0.350
*P*	0.164	0.052	0.221	0.038	0.638	0.993	0.019		0.646	0.599	0.039
*n*	144	144	144	144	144	144	144		36	36	36
DHEAS											
*r*_*p*_	0.027	0.324	0.172	0.450	0.411	0.032	0.028	−0.059		0.186	−0.108
*P*	0.750	0.000	0.040	0.000	0.000	0.705	0.739	0.483		0.285	0.537
*n*	144	144	144	144	144	144	144	144		36	36
LH											
*r*_*p*_	−0.093	−0.045	−0.217	0.028	0.042	0.149	−0.016	0.105	−0.117		−0.103
*P*	0.273	0.594	0.009	0.736	0.618	0.077	0.851	0.214	0.165		0.555
*n*	144	144	144	144	144	144	144	144	144		36
PRL											
*r*_*p*_	0.094	−0.050	0.087	−0.142	−0.058	**−0.199**	0.031	0.037	−0.087	0.072	
*P*	0.270	0.553	0.300	0.091	0.492	**0.017**	0.715	0.659	0.304	0.394	
*n*	144	144	144	144	144	144	144	144	144	144	

*P = 0.022; ^†^ *P* = 0.033 (*r*-to-*z* method, validated as *P* = 0.032 and *P* = 0.029, resp., in the MRA models, as specified in Section 2.3).

**Table 4:** Age- and sex-adjusted correlations for total pre-RA (top) and CN (bottom) log values (imputed in females).

	E2/test	OH-PREG	OH-P4	DHEA	ADIONE	Test	E2	Cortisol	DHEAS	LH	PRL
E2/test											
*r*_*p*_		0.177	0.402	0.065	0.242*	−0.536	0.757	0.160	0.084	−0.29	−0.142
*P*		0.214	0.003	0.651	**0.087**	0.000	0.000	0.262	0.556	0.039	0.32
*n*		54	54	54	54	54	54	54	54	54	54
OH-PREG											
*r*_*p*_	−0.059		0.612	0.716	0.545	0.135	0.218	0.321	0.280	−0.047	0.051
*P*	0.389		0.000	0.000	0.000	0.338	0.121	0.020	0.045	0.739	0.717
*n*	216		54	54	54	54	54	54	54	54	54
OH-P4											
*r*_*p*_	0.188	0.681		0.333	0.452	−0.134	0.350	−0.055	0.064	−0.294	0.094
*P*	0.006	0.000		0.016	0.001	0.343	0.011	0.697	0.654	0.034	0.507
*n*	216	216		54	54	54	54	54	54	54	54
DHEA											
*r*_*p*_	−0.191	0.608	0.373		0.664	0.319	0.138	0.416	0.492	−0.017	−0.157
*P*	0.005	0.000	0.000		0.000	0.021	0.328	0.002	0.000	0.902	0.267
*n*	216	216	216		54	54	54	54	54	54	54
ADIONE											
*r*_*p*_	−**0.090**	0.460	0.396	0.724		0.213	0.418	0.292	0.338	0.062	−0.119
*P*	**0.191**	0.000	0.000	0.000		0.129	0.002	0.036	0.014	0.662	0.399
*n*	216	216	216	216		54	54	54	54	54	54
Test											
*r*_*p*_	−0.605	0.315	0.116	0.321	0.293		−0.104	0.216	−0.110	0.188	−0.042
*P*	0.000	0.000	0.091	0.000	0.000		0.464	0.124	0.439	0.181	0.769
*n*	216	216	216	216	216		54	54	54	54	54
E2											
*r*_*p*_	0.525	0.177	0.354	0.091	0.208	0.098		0.139	0.023	−0.344	−0.061
*P*	0.000	0.009	0.000	0.187	0.002	0.152		0.324	0.870	0.013	0.670
*n*	216	216	216	216	216	216		53	54	54	54
Cortisol											
*r*_*p*_	−0.094	0.308	0.027	0.300	0.236	0.054	−0.118		0.055	−0.057	−0.159
*P*	0.172	0.000	0.698	0.000	0.001	0.433	0.086		0.696	0.687	0.262
*n*	216	216	216	216	216	216	216		54	54	54
DHEAS											
*r*_*p*_	0.037	0.163	0.097	0.438	0.317	−0.047	0.047	−0.044		0.121	−0.053
*P*	0.590	0.017	0.158	0.000	0.000	0.492	0.493	0.520		0.392	0.708
*n*	216	216	216	216	216	216	216	216		54	54
LH											
*r*_*p*_	−0.092	−0.004	−0.186	0.060	0.043	0.094	−0.055	0.118	−0.107		−0.063
*P*	0.180	0.952	0.006	0.386	0.532	0.170	0.423	0.084	0.117		0.656
*n*	216	216	216	216	216	216	216	216	216		54
PRL											
*r*_*p*_	0.082	−0.078	0.007	−0.061	−0.063	−0.211	0.002	0.034	0.072	0.071	
*P*	0.232	0.254	0.923	0.378	0.361	0.002	0.980	0.617	0.294	0.302	
*n*	216	216	216	216	216	216	216	216	216	216	

*P = 0.034 (*r*-to-*z* method, validated as *P* = 0.015, in the MRA model, as specified in Section 2.3).

**Table 5:** Summary of correlations of pre-RA versus CN within each sex group (upper section) and of males versus females within each study group (lower section)*.

Partial correlations observed to differences between preRA versus CN	PreRA versus CN correlations in males (Table 2)						PreRA versus CN correlations in females (Table 3)						*Z*-score diff.	*P* value
	preRA (*n* = 18)		CN (*n* = 72)		ΔCN-preRA		preRA (*n* = 36)		CN (*n* = 144)		ΔCN-preRA	
	*r*_*p*_	*P*	*r*_*p*_	*P*	*Z*	*P*	*r*_*p*_	*P*	*r*_*p*_	*P*	*Z*	*P*
OH-Preg × Cortisol	0.843	**<**0.000	0.557	**<**0.000	**−2.117**	**0.034**	0.075	0.667	0.163	0.052	0.462	0.664^†^	−2.579	0.010
Adione × E2/T ratio	−0.377	0.136	0.051	0.670	1.571	0.116	0.323	0.063	−0.113	0.181	**−2.287**	0.022^‡^	3.858	0.000
Prolactin × T	−0.327	0.200	−0.211	0.078	0.440	0.660	0.207	0.232	−0.199	0.017	**−2.129**	0.033^†^	2.569	0.010

Partial correlations observed to differ between men versus women	Correlations by sex in 54 PreRA (Tables 2 and 3)						Correlations by sex in 216 CN (Tables 2 and 3)							
	Male (*n* = 18)		Female (*n* = 36)		ΔM − F		Male (*n* = 72)		Female (*n* = 144)		ΔM − F			
	*r*_*p*_	*P*	*r*_*p*_	*P*	*Z*	*P*	*r*_*p*_	*P*	*r*_*p*_	*P*	*Z*	*P*		

Adione × E2/T ratio	−0.377	0.136	0.323	0.063	**−2.338**	**0.019**	0.051	0.670	−0.113	0.181	1.117	0.264^‡^	−3.455	*0.0006 *
Prolactin × E2/T ratio	0.404	0.108	−0.241	0.170	**2.115**	**0.031**	0.172	0.152	0.094	0.270	0.539	0.590	1.576	0.1150
LH × DHEAS	−0.424	0.090	0.186	0.285	**−2.058**	**0.040**	0.008	0.947	−0.117	0.165	0.854	0.393^†^	−2.912	*0.0036 *
OH-Preg × cortisol	0.843	**<**0.001	0.075	0.667	3.713	**<**0.001	0.557	**<**0.001	0.163	0.052	3.158	0.002	0.555	0.5789
OH-Preg × DHEAS	0.076	0.773	0.395	0.019	−1.097	0.273	−0.090	0.456	0.324	**<**0.001	**−2.902**	**0.004**	1.805	0.0711
OH-Prog × adione	0.559	0.020	0.439	0.008	0.515	0.607	0.611	**<**0.001	0.329	**<**0.001	**2.510**	0.012*	−1.995	*0.0460 *
OH-Prog × T	0.539	0.026	−0.397	0.018	3.285	0.001	0.508	**<**0.001	−0.074	0.378	4.316	**<**0.001	−1.031	0.3025
OH-Prog × E2/T ratio	−0.355	0.162	0.473	0.005	−2.828	0.005	−0.281	0.018	0.229	0.006	−3.544	**<**0.001	0.716	0.4740
OH-Prog × cortisol	0.496	0.043	−0.239	0.167	2.530	0.011	0.433	**<**0.001	−0.103	0.221	3.859	**<**0.001	−1.329	0.1838
OH-Prog × DHEAS	−0.227	0.380	0.220	0.204	−1.460	0.144	−0.151	0.209	0.172	0.040	**−2.218**	**0.027**	0.758	0.4485
OH-Prog × LH	0.063	0.811	−0.326	0.056	1.289	0.197	0.261	0.028	−0.217	0.009	**3.319**	0.001*	−2.030	*0.0424 *
Prolactin × DHEAS	−0.040	0.879	−0.108	0.537	0.220	0.826	0.416	**<**0.001	−0.087	0.304	**3.608**	**<0.001** ^‡^	−3.388	*0.0007 *
DHEA × cortisol	0.737	0.001	0.224	0.195	2.299	0.021	0.538	**<**0.001	0.173	0.038	2.904	0.004	−0.605	0.5452
Adione × cortisol	0.471	0.057	0.199	0.251	0.994	0.320	0.638	**<**0.001	0.040	0.638	**4.865**	**<0.001** ^‡^	−3.871	*0.0001 *
Adione × DHEAS	−0.207	0.426	0.632	**<**0.001	−3.066	0.002	0.132	0.274	0.411	**<**0.001	−2.069	0.039	−0.997	0.3188
Adione × E2	0.129	0.621	0.502	0.002	−1.356	0.175	0.443	**<**0.001	0.173	0.039	**2.050**	0.040^‡^	−3.406	*0.0007 *
T × E2	0.228	0.379	−0.191	0.272	1.366	0.172	0.297	0.012	0.017	0.839	**1.969**	**0.049**	−0.603	0.5465
Cortisol × E2	0.404	0.108	0.115	0.518	1.005	0.315	0.256	0.031	−0.196	0.019	**3.134**	0.002*	−2.129	*0.0333 *

*Within each sex (upper section), significant differences in partial correlations (Z and *P* values) between pre-RA versus CN (Tables 2 and 3) observed in either pre-RA or CN group, but not in both, are bolded. Within each study group (lower section), only exclusive sex differences (Tables 2 versus Table 3) are bolded.

Two additional low-level sex differences in partial correlations were observed in the total subjects (mentioned in text).

*P < 0.050, >0.010; ^†^ *P* < 0.010, >0.001; ^‡^ *P* < 0.001 probability levels for sex differences (ΔM − F) between study groups (ΔCN − RA).

Differences between *Z* scores in the right minus left panel entries and their corresponding *P* values are listed in the last two columns.

The signs of the Δ*Z*-scores derive from subtraction of the *Z* values in the right panel (females in upper and CN in lower row entries) from the *Z* values in the left panel (males in upper and pre-RA in lower entries), respectively (see text).
